# Stress, Anxiety, and Depression Among Spanish University Professors: Associations With Lifestyle Habits and Physical and Mental Health Indicators

**DOI:** 10.62641/aep.v54i1.2054

**Published:** 2026-02-15

**Authors:** María Estela Colado Tello, Esther Gargallo Ibort, Josep Maria Dalmau Torres, Raúl Jiménez Boraita

**Affiliations:** ^1^Department of Education Sciences, University of La Rioja, 26004 Logroño, La Rioja, Spain; ^2^Department of Health Sciences, International University of La Rioja, 26006 Logroño, La Rioja, Spain

**Keywords:** health, lifestyle, mental disorders, university, stress, anxiety

## Abstract

**Background:**

Professors play a crucial role in the educational process, making their well-being a key area of interest in research on universities as health-promoting settings. The scientific literature emphasizes that various contextual, personal, and behavioral factors have a direct impact on faculty health. To estimate the prevalence of stress, anxiety, and depression among Spanish university professors, and to examine their associations with lifestyle habits and indicators of physical and mental health.

**Methods:**

A cross-sectional study was conducted with 1560 participants (mean age 47.39 ± 11.29 years) from thirteen universities that are part of the Spanish Network of Health-Promoting Universities. The variables assessed included stress, anxiety, depression, burnout, health-related quality of life, physical activity, sedentary behaviour, dietary patterns, sleep quality, and vocal fatigue.

**Results:**

Regression analyses revealed that, across all three outcomes, lower mental well-being, greater emotional exhaustion, and more frequent sleep disturbances were significant predictors of psychological distress. For stress and anxiety, being female and younger also emerged as significant demographic predictors. Stress was additionally associated with increased emotional eating and reduced vocal recovery, whereas anxiety was linked to greater physical vocal discomfort. Depression was predicted exclusively by lower mental well-being, higher emotional exhaustion, and more sleep problems.

**Conclusions:**

The psychological health of university faculty is influenced by a complex interplay of well-being, occupational, and lifestyle factors. Interventions aimed at enhancing emotional regulation, promoting sleep hygiene, ensuring balanced workloads, and providing psychosocial support, along with institutional measures that address early-career vulnerabilities and gender disparities, may help mitigate stress, anxiety, and depression among university professors.

## Introduction

Universities are institutions dedicated to the creation and dissemination of knowledge through research and teaching, in which faculty members act as key agents. As such, their health is a central focus in research exploring universities as health-promoting environments [1, 2]. In this regard, various studies have examined the health of both students and faculty, concluding that multiple contextual, personal, and behavioural factors such as workload, pace of work, time pressures, friction with colleagues, and relational dynamics with students have a direct impact on health [3, 4, 5, 6]. Furthermore, research conducted in various countries has identified that the teaching profession significantly affects the mental well-being of those who practice it, revealing that teachers are particularly vulnerable compared to other professionals to experiencing high levels of stress, anxiety, and depression [7, 8, 9, 10].

In 2022, the WHO reported that approximately 12.5% of the global population were living with a mental disorder [11]. Furthermore, the COVID-19 pandemic was associated with an estimated 25% increase in the prevalence of anxiety and depression globally [12]. This increase led to a documented worsening of psychiatric symptoms. These mental health issues have also extended to the academic setting, with numerous reports highlighting increasingly prominent stressors and emotional effects among university teaching staff [4]. While the prevalence of stress, anxiety, and depression prior to the pandemic has been extensively studied in non-university educational contexts and non-clinical populations, the university setting has received comparatively less attention [13]. Nonetheless, existing studies indicate that stress-related problems are common among university professors, although results vary considerably between studies [14, 15, 16].

University faculty face constant demands regarding research productivity, keeping teaching content updated, and navigating challenging working conditions. These pressures increase their vulnerability to psycho-emotional and psychosomatic disorders derived from occupational demands [17, 18]. Previous studies have emphasized the need to address excessive stress among university professors, as it can trigger a series of serious consequences and increase the risk of somatic and mental health issues such as burnout and psychological distress [19, 20]. Moreover, a significant relationship has been found between mental health and quality of life in educators, although this has been mostly documented among non-university teaching staff [21].

Work-related stressors have also been associated with vocal symptoms in teachers, which negatively affect professional performance and increase the risk of vocal problems [22, 23]. Muscular tension resulting from stress, anxiety, and depression can impair vocal function, leading to dysphonia, vocal cord disorders, and diminished vocal quality [24]. Evidence also shows that teachers with vocal disorders are more likely to experience stress and anxiety [25].

In parallel, lifestyle habits play a critical role in the health of university faculty [26]. The lack of work-life balance, long working hours, and irregular schedules can lead to physical and emotional exhaustion, thereby exacerbating anxiety and depression [4]. Furthermore, the tendency to sacrifice time for self-care such as engaging in regular physical activity and ensuring adequate sleep can negatively impact mental health [27]. Research confirms associations between dietary habits, physical activity, and sleep quality, identifying these factors as determinants of stress, anxiety, and depression levels in the general population [28, 29, 30].

The current literature highlights that mental health challenges affect all levels of the academic hierarchy, from trainee teachers to university professors. Depression, anxiety, and stress have a considerable impact on the teaching profession, and the university context is no exception [4, 12]. However, most research has focused on educators at non-university stages, with relatively few comprehensive analyses of mental health in university faculty [4, 12]. Furthermore, evidence suggests that the mental well-being of university professors may vary according to sociodemographic variables such as gender, age, years of experience, and academic rank [4]. Several studies indicate that women in academia tend to experience higher levels of stress, anxiety, and burnout than men, a trend observed in various university contexts [14, 31]. Similarly, younger faculty or those in the early stages of their careers may show greater emotional vulnerability, possibly due to lower job stability, limited coping resources, and increased performance expectations during the early years of professional development [32, 33, 34].

Based on previous evidence, it was anticipated that health-promoting behaviours such as higher levels of physical activity, better sleep quality, and healthier eating patterns would be negatively associated with symptoms of stress, anxiety, and depression [29, 30]. Conversely, maladaptive behaviours including prolonged sedentary time, sleep disturbances, and emotional eating were expected to be positively associated with greater psychological distress [30]. These expectations are supported by a substantial body of research identifying lifestyle habits as key determinants of mental health in both general and teaching populations [27, 28, 29, 30]. Furthermore, the inclusion of variables such as physical activity, sedentary behaviour, dietary habits, sleep quality, and vocal fatigue is justified given their documented relevance in the university teaching population, whose demanding work conditions impact both physical and psychological well-being. Clarifying these hypothesised associations reinforces the alignment between the theoretical framework, the study design, and the interpretation of the findings. Therefore, the objective of the present study was to determine the prevalence of stress, anxiety, and depression among Spanish university faculty and examine their relationship with lifestyle habits and indicators of physical and mental health. Accordingly, the study assessed stress, anxiety, depression, quality of life, burnout, vocal fatigue, physical activity, sedentary behaviour, dietary patterns, and sleep quality.

## Material and Methods

### Study Design and Participants 

A cross-sectional, correlational descriptive design was applied. Data were collected using an online questionnaire. Participants were recruited through convenience sampling from university faculty members affiliated with institutions belonging to the Spanish Network of Health-Promoting Universities. The final sample consisted of 1560 university professors from thirteen institutions. Baseline demographic and professional characteristics of the sample are shown in Table 1.

**Table 1.** **Baseline demographic and professional characteristics of the sample**.

Characteristic		Sample (N = 1560)
Gender, n (%)		
	Female	781 (50.1)
	Male	779 (49.9)
Age (years), mean ± SD (range)		47.39 ± 11.29 (23–74)
Years of teaching experience, mean ± SD (range)		16.35 ± 11.93 (0–51)
Academic rank, n (%)		
	Full or associate professor	694 (44.5)
	Teaching faculty (contracted and assistant lecturers)	424 (27.2)
	Adjunct faculty	257 (16.5)
	Research staff with teaching responsibilities	182 (11.7)

SD, standard deviation.

### Procedure

The study was conducted in accordance with the ethical standards outlined in the Declaration of Helsinki and received prior approval from the Research Ethics Committee of the University of La Rioja (approval code: vp0TQWiHARguHNsNbDiWiaaGFXAHhs3A). All participants provided informed consent before completing the questionnaire. Participants were invited via their institutional email accounts to take part in the survey. They were informed about the objectives of the research and asked to provide consent before accessing the questionnaire. Participation was entirely voluntary and anonymous. The survey was distributed using institutional email from the collaborating universities, accompanied by an introduction to the study and a link to the SurveyMonkey platform. Respondents were given a 90-day window to complete the survey, and two reminder emails containing the survey link were sent to those who had not yet responded. Data collection took place between November 2023 and January 2024.

### Instruments

Before completing the psychological and lifestyle measures, participants responded to a brief sociodemographic form that included items on age, gender, academic position (associate, assistant, lecturer, senior lecturer, full professor), and years of teaching experience. This information was used to characterize the overall profile of the university teaching staff in the sample.

Mental health disorders were assessed using the Depression, Anxiety, and Stress Scale (DASS-21), developed by Lovibond and Lovibond [35] and validated for Spanish populations by Fonseca-Pedrero *et al*. [36]. This instrument evaluates the intensity of negative emotional states experienced during the previous week through 21 items rated on a four-point Likert scale (0 = did not apply to me, 3 = applied to me very much or most of the time). The scale comprises three subscales (depression, anxiety, and stress), each with seven items. Subscale scores are calculated by summing the relevant responses and multiplying the raw scores by two to ensure comparability with both the DASS-21 and DASS-42 formats, yielding a range from 0 to 42. Higher scores indicate greater severity of stress, anxiety, or depression. In the present study, Cronbach’s alpha coefficients were 0.894 for stress, 0.791 for anxiety, and 0.907 for depression, reflecting excellent internal consistency for the stress and depression subscales and acceptable consistency for the anxiety subscale. The following cut-off points were applied [37]: stress (0–14 = normal, 15–25 = mild/moderate, ≥26 = severe/extremely severe); anxiety (0–7 = normal, 8–15 = mild/moderate, ≥16 = severe/extremely severe); and depression (0–9 = normal, 10–20 = mild/moderate, ≥21 = severe/extremely severe).

Burnout was assessed using the Spanish adaptation of the Maslach Burnout Inventory Educators Survey (MBI-ES), originally developed by Maslach and Jackson [38] and validated for Spanish teachers by Ferrando and Pérez [39]. This instrument includes 22 items rated on a seven-point Likert scale ranging from 0 (never) to 6 (every day). It consists of three subscales: emotional exhaustion, depersonalisation, and personal accomplishment. Subscale scores are obtained by summing the corresponding items. Burnout is indicated by high scores in emotional exhaustion and depersonalisation, and low scores in personal accomplishment. Cronbach’s alpha coefficients in the current study were 0.919 for emotional exhaustion, 0.740 for depersonalisation, and 0.906 for personal accomplishment, reflecting excellent reliability for the first and third subscales, and acceptable reliability for the second.

Health-related quality of life was measured using the WHOQOL-BREF, the abbreviated version of the World Health Organization Quality of Life scale [40]. This tool evaluates self-perceptions of health, psychosocial functioning, and related quality of life aspects over the previous two weeks. It includes 26 items rated on a five-point Likert scale and grouped into four domains: physical health (seven items), psychological health (six items), social relationships (three items), and environmental health (eight items). Two additional items assess overall quality of life and general health. Raw domain scores can be transformed into a 0–100 scale, with higher values indicating better quality of life. Cronbach’s alpha coefficients were 0.754 for physical health, 0.834 for psychological health, 0.744 for social relationships, and 0.807 for environmental health, indicating acceptable to good internal consistency across domains.

Physical activity was assessed using the International Physical Activity Questionnaire Short Form (IPAQ-SF), validated in 12 countries including Spain [41]. This instrument gathers data on the intensity, frequency, and duration of physical activity during the past seven days. It includes seven items regarding vigorous activity, moderate activity, and walking, along with time spent on each. Weekly physical activity was estimated in metabolic equivalents of task (MET)-minutes per week in accordance with the IPAQ scoring protocol [42]. The main outcome was total physical activity expressed in MET-minutes.

Sedentary behaviour was measured with the short form of the Sedentary Behaviour Questionnaire (SBQ-S), developed by Rosenberg *et al*. [43] and validated in Spain by Munguía-Izquierd *et al*. [44], with intraclass correlation coefficients between 0.83 and 0.86. The questionnaire consists of 11 items assessing time spent in sedentary activities on weekdays and weekends (e.g., watching television, listening to music). Responses are provided on a nine-point scale ranging from ‘nothing’ to ‘six hours or more’. Weekly sedentary time is computed by summing scores across all items, allowing for differentiation between weekday and weekend behaviours.

Dietary habits were evaluated using the Spanish version of the Three-Factor Eating Questionnaire-R18 (TFEQ-SP), a shortened form of the original instrument by Stunkard and Messick [45], validated by Jáuregui-Lobera *et al*. [46]. This tool assesses three domains: (a) cognitive restraint (six items); (b) disinhibition or uncontrolled eating (nine items); and (c) emotional eating (three items). Participants respond on a four-point Likert scale ranging from ‘totally true’ to ‘totally untrue’. Scores are calculated for each domain, with higher scores reflecting stronger tendencies toward each respective behaviour. In this study, Cronbach’s alpha coefficients were 0.775 for cognitive restraint, 0.858 for uncontrolled eating, and 0.837 for emotional eating, reflecting acceptable to good reliability.

Sleep quality was assessed using the Pittsburgh Sleep Quality Index (PSQI), developed by Buysse *et al*. [47] and adapted for Spanish populations by Royuela and Macías [48]. The PSQI evaluates subjective sleep quality and disturbances during the previous month. It includes 19 items grouped into seven components: subjective sleep quality, sleep latency, sleep duration, habitual sleep efficiency, sleep disturbances, use of sleep medication, and daytime dysfunction. Each component is scored from 0 to 3. A global score, ranging from 0 to 21, is obtained by summing component scores, with higher values indicating poorer sleep quality. The Cronbach’s alpha for the PSQI in this study was 0.701, indicating acceptable internal consistency.

Vocal fatigue was measured using the Voice Fatigue Index (VFI), developed by Nanjundeswaran *et al*. [49] and validated in Spanish teachers by Contreras-Regatero *et al*. [50]. This 19-item questionnaire captures symptoms related to vocal fatigue associated with voice use. It includes three factors: voice tiredness and avoidance (11 items), physical discomfort (5 items), and symptom improvement with rest (3 items). Responses are rated on a five-point scale ranging from 0 (never) to 4 (always). In this study, Cronbach’s alpha values were 0.942 for voice tiredness and avoidance, 0.921 for physical discomfort, and 0.942 for symptom improvement with rest, all indicating excellent internal consistency. Subscale scores are obtained by summing relevant item responses. High scores in factors 1 and 2 reflect greater vocal fatigue, while higher scores in factor 3 indicate symptom relief following rest.

### Statistical Analysis

Quantitative variables were described using means and standard deviations. The distribution of all variables was examined using the Kolmogorov–Smirnov test, and normality was assessed prior to selecting the appropriate statistical tests. Levene’s test was applied to assess homoscedasticity. Depending on the fulfilment of these assumptions, group comparisons across stress, anxiety, and depression categories were performed using one-way ANOVA (for normally distributed data with homogeneous variances) or the Kruskal–Wallis H test (for non-normally distributed or heteroscedastic data).

Bivariate associations between stress, anxiety, depression, and all lifestyle and health-related variables were examined using correlation coefficients. Pearson’s correlation was used for variables with normal distribution, while Spearman’s rho was applied when the assumption of normality was not met.

To identify the variables associated with stress, anxiety, and depression, multiple linear regression models were estimated using a backward elimination procedure. The initial models included demographic variables (gender, age, and years of teaching experience), along with all lifestyle and health-related variables assessed in the study. These included: physical, mental, environmental, and social well-being; emotional exhaustion, depersonalisation, and personal accomplishment; physical activity and sedentary behaviour; cognitive restraint, disinhibited eating, and emotional eating; sleep issues; and vocal tiredness, physical discomfort, and symptom improvement with rest. For the multiple regression models, stress, anxiety, and depression were treated as continuous variables, using the total scores from the corresponding DASS-21 subscales. Multicollinearity was assessed using Tolerance values and the Variance Inflation Factor (VIF). All VIF values ranged from 1.04 to 1.71, indicating no problematic multicollinearity. All statistical analyses were conducted using IBM SPSS Statistics for Windows, version 25.0 (IBM Corp., Armonk, NY, USA), with statistical significance set at *p* < 0.05.

## Results 

Baseline sociodemographic and professional characteristics of the participants are presented in Table 1. The sample consisted of 1560 Spanish university professors from thirteen institutions, of whom 50.1% were women and 49.9% were men. The mean age was 47.39 ± 11.29 years (range: 23–74), and the mean university teaching experience was 16.35 ± 11.93 years (range: 0–51). Regarding academic rank, 44.5% of the participants were full or associate professors, 27.2% belonged to the teaching faculty category (including contracted and assistant lecturers), 16.5% were adjunct faculty, and 11.7% were research staff with teaching responsibilities. Based on the DASS-21 cut-off scores, 24.55% of the participants presented mild/moderate or severe/extremely severe stress symptoms, 29.55% showed elevated anxiety symptoms, and 29.78% reported elevated depressive symptoms.

Indicators of quality of life, burnout, and vocal fatigue were analyzed in relation to levels of stress, anxiety, and depression (Table 2). Overall, university professors with mild/moderate or severe/extremely severe symptoms reported significantly lower physical, psychological, environmental, and social well-being (*p* < 0.001), along with higher levels of emotional exhaustion and depersonalization (*p* < 0.001), and increased vocal fatigue, particularly tiredness and physical discomfort, compared to those within the normal range (*p* < 0.001). Personal accomplishment was also lower among professors experiencing more severe psychological symptoms (*p* < 0.001), although this decline did not follow a clearly linear pattern across stress levels. Additionally, improvement in vocal symptoms varied significantly only in relation to stress levels (*p* = 0.001), with no significant differences observed across categories of anxiety (*p* = 0.455) or depression (*p* = 0.130).

**Table 2.** **Quality of life, burnout and vocal fatigue as a function of stress, anxiety and depression**.

	Stress			Anxiety			Depression		
	Normal	Slight/Moderate	Severe/Extr. Severe	Normal	Slight/Moderate	Severe/Extr. Severe	Normal	Slight/Moderate	Severe/Extr. Severe
	N = 1177	N = 262	N = 121	N = 1204	N = 251	N = 105	N = 1202	N = 264	N = 94
Physical wellbeing	76.06 ± 13.29	61.71 ± 14.93	55.96 ± 16.37	75.60 ± 13.65	62.81 ± 14.55	54.05 ± 16.58	75.99 ± 13.38	61.13 ± 14.81	53.04 ± 14.49
	<0.001			<0.001			<0.001		
Mental wellbeing	72.32 ± 13.89	55.10 ± 16.60	45.70 ± 18.95	71.73 ± 14.44	55.68 ± 16.81	45.24 ± 18.82	73.20 ± 12.96	51.97 ± 13.42	35.90 ± 15.17
	<0.001			<0.001			<0.001		
Environmental wellbeing	72.15 ± 13.85	62.70 ± 15.37	57.31 ± 17.45	72.16 ± 13.95	61.91 ± 14.44	55.92 ± 17.95	72.42 ± 13.67	61.55 ± 14.29	53.09 ± 18.45
	<0.001			<0.001			<0.001		
Social wellbeing	65.96 ± 19.24	55.34 ± 21.24	49.93 ± 22.61	65.60 ± 19.37	55.44 ± 21.45	50.24 ± 22.95	67.27 ± 18.32	51.52 ± 20.08	39.54 ± 21.55
	<0.001			<0.001			<0.001		
Emotional exhaustion	14.64 ± 8.92	26.46 ± 10.70	32.94 ± 12.46	15.38 ± 9.73	24.43 ± 10.75	33.30 ± 12.01	15.05 ± 9.24	25.86 ± 10.90	34.33 ± 12.90
	<0.001			<0.001			<0.001		
Depersonalisation	2.33 ± 2.98	3.94 ± 3.95	4.58 ± 5.25	2.45 ± 3.11	3.39 ± 3.78	4.98 ± 5.28	2.31 ± 2.95	3.78 ± 3.84	5.89 ± 5.76
	<0.001			<0.001			<0.001		
Personal accomplishment	33.17 ± 8.92	30.61 ± 8.33	31.07 ± 9.81	33.09 ± 8.87	31.38 ± 9.00	29.50 ± 8.95	33.35 ± 8.76	30.27 ± 8.69	29.20 ± 10.22
	<0.001			<0.001			<0.001		
Vocal tiredness	8.28 ± 7.93	13.10 ± 9.83	14.24 ± 11.83	8.18 ± 7.93	13.00 ± 9.44	17.18 ± 11.80	8.62 ± 8.24	11.84 ± 9.30	15.19 ± 12.36
	<0.001			<0.001			<0.001		
Physical vocal discomfort	2.34 ± 3.36	4.31 ± 4.33	4.74 ± 5.29	2.24 ± 3.22	4.55 ± 4.59	5.93 ± 5.30	2.54 ± 3.57	3.53 ± 3.99	5.07 ± 5.32
	<0.001			<0.001			<0.001		
Vocal symptom improvement	4.95 ± 4.02	5.36 ± 3.70	3.80 ± 2.99	4.91 ± 4.17	5.17 ± 3.65	4.67 ± 3.23	5.07 ± 4.01	4.68 ± 3.63	3.90 ± 3.16
	0.001			0.455			0.130		

Table 3 displays the results related to physical activity, sedentary time, sleep issues, and eating behaviour according to levels of stress, anxiety, and depression. Reduced physical activity levels were observed among university professors with mild/moderate and severe/extremely severe symptoms of stress (*p* = 0.001) and anxiety (*p* = 0.002), whereas no significant differences were found in relation to depression levels (*p* = 0.526). Sedentary time was significantly higher in groups with greater symptom severity across all three psychological conditions (stress: *p* = 0.001; anxiety: *p* = 0.042; depression: *p* = 0.005). Sleep issues also recorded elevated scores among professors experiencing more severe stress, anxiety, or depression (*p* < 0.001). Regarding eating behaviour, both disinhibited eating and emotional eating were more common among those with higher levels of stress, anxiety, or depression (*p* < 0.001). In contrast, levels of cognitive restraint did not differ significantly across degrees of stress, anxiety, or depression.

**Table 3.** **Physical activity engagement, sedentary time and eating behaviour as a function of stress, anxiety and depression**.

	Stress			Anxiety			Depression		
	Normal	Slight/Moderate	Severe/Extr. Severe	Normal	Slight/Moderate	Severe/Extr. Severe	Normal	Slight/Moderate	Severe/Extr. Severe
	N = 1177	N = 262	N = 121	N = 1204	N = 251	N = 105	N = 1202	N = 264	N = 94
METs	2593.11 ± 2290.89	2189.60 ± 1886.22	1974.42 ± 1769.08	2566.13 ± 2241.49	2204.49 ± 1995.59	2111.58 ± 2111.36	2547.90 ± 2242.74	2290.17 ± 1866.95	2546.09 ± 2295.65
	0.001			0.002			0.526		
Sedentary time	3509.62 ± 1021.31	3742.44 ± 1072.15	3822.31 ± 1241.97	3530.44 ± 1032.10	3665.62 ± 1010.60	3839.19 ± 1326.46	3518.26 ± 1017.72	3749.09 ± 1079.60	3777.98 ± 1333.39
	0.001			0.042			0.005		
Sleep issues	4.22 ± 2.32	5.87 ± 2.54	6.85 ± 3.15	4.26 ± 2.32	5.85 ± 2.70	7.03 ± 2.99	4.30 ± 2.35	5.84 ± 2.77	6.65 ± 2.95
	<0.001			<0.001			<0.001		
Cognitive restraint	15.70 ± 4.29	15.50 ± 4.39	16.22 ± 4.83	15.67 ± 4.32	15.73 ± 4.30	16.07 ± 4.86	15.63 ± 4.31	15.81 ± 4.43	16.47 ± 4.65
	0.378			0.836			0.241		
Disinhibited eating	15.71 ± 4.50	17.37 ± 5.29	19.29 ± 5.94	15.72 ± 4.56	17.87 ± 5.15	18.68 ± 6.09	15.81 ± 4.58	17.45 ± 5.32	18.73 ± 5.97
	<0.001			<0.001			<0.001		
Emotional eating	5.26 ± 2.15	6.34 ± 2.42	7.36 ± 2.85	5.32 ± 2.22	6.35 ± 2.32	7.12 ± 2.83	5.29 ± 2.15	6.47 ± 2.51	7.28 ± 2.89
	<0.001			<0.001			<0.001		

METs, metabolic equivalents of task.

Table 4 summarizes the correlations between the study variables and levels of stress, anxiety, and depression. Overall, the analysis revealed consistent and comparable patterns across the three psychological conditions. First, stress, anxiety, and depression were negatively and significantly associated with all dimensions of quality of life, including physical, mental, environmental, and social well-being, as well as with personal accomplishment (*p* < 0.01). In contrast, these three psychological indicators showed positive and significant correlations with emotional exhaustion, depersonalization, sedentary time, sleep issues, vocal tiredness, and physical vocal discomfort (*p* < 0.01). Furthermore, regarding eating behaviours, both disinhibited eating and emotional eating were positively and significantly related to higher levels of stress, anxiety, and depression (*p* < 0.01), whereas cognitive restraint showed no significant correlation with any of these conditions. In addition, physical activity levels (METs) were negatively and significantly associated with stress and anxiety (*p* < 0.01), while the correlation with depression did not reach statistical significance. Finally, improvement in vocal symptoms with rest showed a significant negative correlation exclusively with stress (*p* < 0.05), with no significant associations observed for either anxiety or depression.

**Table 4.** **Correlation coefficients relating indicators of physical and mental health and lifestyle habits with stress, anxiety and depression**.

	Stress	Anxiety	Depression
Physical wellbeing	–0.533^**^	–0.485^**^	–0.518^**^
Mental wellbeing	–0.593^**^	–0.513^**^	–0.716^**^
Environmental wellbeing	–0.397^**^	–0.374^**^	–0.430^**^
Social wellbeing	–0.335^**^	–0.265^**^	–0.469^**^
Emotional exhaustion	0.631^**^	0.525^**^	0.565^**^
Depersonalisation	0.285^**^	0.228^**^	0.304^**^
Personal accomplishment	–0.151^**^	–0.122^**^	–0.176^**^
Vocal tiredness	0.300^**^	0.355^**^	0.255^**^
Physical vocal discomfort	0.265^**^	0.341^**^	0.208^**^
Vocal symptom improvement	–0.059^*^	–0.001	–0.048
METs	–0.096^**^	–0.082^**^	–0.041
Sedentary time	0.120^**^	0.081^**^	0.098^**^
Sleep issues	0.414^**^	0.367^**^	0.330^**^
Cognitive restraint	0.034	0.013	0.037
Disinhibited eating	0.271^**^	0.245^**^	0.213^**^
Emotional eating	0.329^**^	0.273^**^	0.282^**^

Notes: ** *p* < 0.01 (two-tailed); * *p* < 0.05 (two-tailed); Correlation coefficients correspond to Pearson’s *r* for variables meeting normality assumptions and Spearman’s rho for those that did not. METs, metabolic equivalents of task.

Table 5 displays the results of the multiple linear regression models for stress, anxiety, and depression. Gender, age, and years of teaching experience were included as covariates in the models. Regarding stress, the model showed that male gender (B = –1.241, *p* < 0.001) and younger age (B = –0.125, *p* < 0.001) were significantly associated with lower stress scores. In addition, lower levels of mental well-being (B = –0.139, *p* < 0.001), higher emotional exhaustion (B = 0.285, *p* < 0.001), more frequent sleep disturbances (B = 0.644, *p* < 0.001), and greater emotional eating (B = 0.205, *p* = 0.005) emerged as significant predictors of increased stress. The model accounted for 53.9 percent of the variance. Similarly, the model predicting anxiety indicated that male gender (B = –0.580, *p* = 0.017) and younger age (B = –0.060, *p* < 0.001) were associated with lower anxiety levels, again suggesting higher anxiety among women. Lower mental well-being (B = –0.092, *p* < 0.001), greater emotional exhaustion (B = 0.127, *p* < 0.001), increased sleep disturbances (B = 0.357, *p* < 0.001), and higher physical vocal discomfort (B = 0.270, *p* < 0.001) also emerged as significant predictors. This model explained 40.7 percent of the variance. Finally, in the model predicting depression, lower mental well-being (B = –0.260, *p* < 0.001), higher emotional exhaustion (B = 0.154, *p* < 0.001), and more frequent sleep disturbances (B = 0.160, *p* < 0.001) were identified as significant predictors. This model accounted for 54.9 percent of the variance.

**Table 5.** **Regression outcomes in relation to stress, anxiety and depression**.

	B	SD	Std. B	t	*p*	VIF	R^2^
Stress							
Gender (Male)	−1.241	0.322	−0.069	−3.856	<0.001	1.083	0.539
Age	−0.125	0.014	−0.157	−8.807	<0.001	1.080	
Mental wellbeing	−0.139	0.012	−0.267	−11.903	<0.001	1.704	
Emotional exhaustion	0.285	0.017	0.360	16.621	<0.001	1.587	
Sleep issues	0.644	0.066	0.185	9.743	<0.001	1.214	
Emotional eating	0.205	0.073	0.053	2.826	0.005	1.209	
Anxiety							
Gender (Male)	−0.580	0.243	−0.048	−2.386	0.017	1.072	0.407
Age	−0.060	0.011	−0.112	−5.532	<0.001	1.082	
Mental wellbeing	−0.092	0.009	−0.264	−10.662	<0.001	1.611	
Emotional exhaustion	0.127	0.013	0.239	9.487	<0.001	1.671	
Sleep issues	0.357	0.050	0.153	7.093	<0.001	1.225	
Physical vocal discomfort	0.270	0.033	0.172	8.217	<0.001	1.151	
Depression							
Mental wellbeing	−0.260	0.010	−0.569	−26.477	<0.001	1.595	0.549
Emotional exhaustion	0.154	0.015	0.222	10.591	<0.001	1.514	
Sleep issues	0.160	0.056	0.052	2.830	<0.001	1.175	

VIF, Variance Inflation Factor; SD, standard deviation.

## Discussion

The present findings reveal significant associations between stress, anxiety, and depression, and various lifestyle habits and indicators of physical and mental health. The observed prevalence rates were 24.55% for stress, 29.55% for anxiety, and 29.78% for depression. These figures exceed those reported in previous studies involving university faculty in countries such as Malaysia and Mexico [51, 52], yet remain lower than those observed during the COVID-19 pandemic in Spain and Brazil [7, 9]. During the pandemic, stressors such as the shift to online teaching and concerns over reopening institutions significantly contributed to teachers’ psychological distress [8, 10]. This study adds to growing evidence that the teaching profession is especially vulnerable to mental health challenges, often more acutely than other professions such as nursing, social work, medicine, or dentistry [53].

The regression models also revealed relevant demographic patterns. Regarding gender, female professors reported higher levels of stress and anxiety than their male counterparts, a finding consistent with previous research conducted among university faculty. Redondo-Flórez *et al*. [16] found that Spanish female academics reported significantly higher levels of perceived stress and emotional exhaustion than male professors, while Garcés-Delgado *et al*. [54] noted that women, particularly those at the beginning of their academic careers, tend to experience a greater burden of work-related stress. Although the mechanisms underlying these differences cannot be definitively established, the literature suggests that factors such as heavier teaching loads, limited access to resources, challenges in balancing professional and personal roles, and certain organizational conditions may differentially affect men and women [5, 16, 54, 55]. Nevertheless, these trends should be interpreted with caution, as some studies have reported heterogeneous patterns depending on the institutional context and academic discipline. Younger faculty members also showed higher levels of psychological distress, a pattern consistent with the existing literature on academic staff in the early stages of their professional trajectories. Hammoudi Halat *et al*. [4] highlight that younger university teachers tend to exhibit greater emotional vulnerability due to the pressure to consolidate their careers, contractual instability, and high demands for productivity. In line with this, recent studies have indicated that the initial stages of an academic career are characterized by elevated levels of stress, anxiety, and depression, primarily associated with job insecurity, precarious working conditions, and limited experience in managing university-related demands [56, 57].

The regression models revealed relevant demographic trends. Regarding gender, female professors reported higher levels of stress and anxiety than their male counterparts, consistent with prior findings among Spanish university faculty. For instance, Redondo-Flórez *et al*. [16] reported greater perceived stress and emotional exhaustion among women, while Garcés-Delgado *et al*. [54] noted that early-career female academics often face a greater burden of occupational stress. Though definitive mechanisms remain unclear, factors such as heavier teaching loads, limited resources, difficulty balancing professional and personal roles, and organizational barriers may contribute to these differences [5, 55]. However, these patterns should be interpreted with caution, given that results can vary based on institutional and disciplinary contexts. Younger faculty members also reported higher levels of psychological distress, in line with existing research on early-career academics. Hammoudi Halat *et al*. [4] emphasize that contractual instability, pressure to publish, and professional inexperience contribute to emotional vulnerability in this group. Supporting this, recent studies have documented that early academic careers are marked by heightened stress, anxiety, and depression, linked to job insecurity and demanding work environments [56, 57].

Regarding quality of life, the mental well-being dimension showed the strongest and most consistent associations with stress, anxiety, and depression. Professors with lower mental well-being reported higher levels of all three conditions, a pattern that aligns with previous research indicating that psychological well-being is closely linked to anxiety, depressive symptoms, and perceived stress [58, 59]. Moreover, higher levels of mental well-being have been proposed as a potential protective factor against the development or recurrence of common mental disorders, and evidence suggests that teachers’ subjective well-being and depressive symptoms are strongly related to stress and anxiety levels [60, 61]. This pattern was clearly reflected in the multivariate analyses of the present study, where mental well-being emerged as a significant predictor of stress, anxiety, and depression.

In addition, the physical, environmental, and social quality of life domains were also significantly associated with stress, anxiety, and depression [62]. For physical well-being, both group comparisons and correlation analyses indicated that professors with higher psychological distress reported poorer perceptions of their physical health, a finding consistent with previous evidence linking mental health problems to reduced physical functioning and health-related quality of life [62, 63]. In particular, anxiety has been frequently linked to chronic physical comorbidities such as cardiovascular disease, diabetes, and respiratory conditions [64, 65, 66]. Environmental well-being was also negatively associated with stress, anxiety, and depression. This finding aligns with research showing that environmental conditions, such as housing quality, access to services, and perceived environmental comfort, are associated with poorer mental health outcomes when perceived as inadequate [67]. Similarly, studies on natural outdoor environments have demonstrated that exposure to green spaces is associated with reduced stress and improved emotional well-being, reinforcing the sensitivity of the environmental domain to psychological distress [68]. Finally, social well-being was also negatively associated with stress, anxiety, and depression. This result is consistent with evidence indicating that low social support and weaker interpersonal cohesion are related to a higher risk of psychological distress, including anxiety and depressive symptoms [69, 70]. Research on social capital further suggests that perceiving one’s social relationships as unsatisfactory or belonging to a less connected social environment is associated with poorer mental health, which is also in line with the present findings [69].

With regard to burnout, the regression analysis revealed positive associations between emotional exhaustion and levels of stress, anxiety, and depression. These results are consistent with previous studies reporting significant correlations between psychological distress and burnout symptoms [16, 71, 72]. The existing literature supports the notion that burnout, anxiety, depression, and stress are intrinsically interconnected [69]. However, it is important to highlight that burnout is conceptualised not as a mental disorder, but as a psychological response pattern triggered by exposure to demanding or aversive work situations [36, 73, 74].

In line with this, previous research involving university faculty has shown that higher levels of psychological symptoms are associated with greater emotional exhaustion and depersonalisation, along with lower personal accomplishment [75]. However, in the present study, the personal accomplishment dimension showed a less clearly defined pattern in relation to stress. This variability is consistent with the conceptual nature of the construct, as personal accomplishment reflects a self-evaluative perception of competence and professional success, rather than an immediate emotional reaction to work stress [74]. Moreover, empirical evidence suggests that the causal patterns among burnout dimensions are not uniform and do not follow a single developmental trajectory. Personal accomplishment may evolve differently from emotional exhaustion and depersonalization [76]. Within this conceptual framework, it is understandable that personal accomplishment did not show a clear linear gradient across stress categories in the present study, in contrast to the other two burnout dimensions.

In relation to vocal fatigue, regression analyses indicated that university professors experiencing higher levels of stress, anxiety, and depression reported greater physical discomfort in their voice. Vocal health may be affected by various factors related to vocal demands, behaviours, environmental elements, and emotional or psychological states [77]. Several studies have shown that depressive symptoms, stress, and anxiety are significant predictors of vocal problems in teaching staff [22, 78]. For example, excessive stress has been directly associated with vocal fatigue, in addition to factors such as dry mouth, upper respiratory infections, or the need to raise one’s voice. Stress has also been identified as a contributing factor in the development of vocal cord disorders [23, 79].

Similarly, some researchers suggest that when stress and tension are present, vocal fatigue and discomfort may emerge due to changes in respiratory patterns, alterations in mucosal membranes due to blood flow or hydration changes, or increased muscular tension [80, 81, 82]. The fact that vocal symptom improvement with rest was observed only in relation to stress, and not anxiety or depression, may be related to the more reactive psychophysiological nature of stress responses. Experimental evidence shows that psychological stressors can elicit transient increases in intrinsic laryngeal muscle activity that return to baseline once the stressor is removed [83]. In occupational and clinical contexts, stress has also been associated with laryngeal muscle tension and vocal [84]. In parallel, studies on vocal loading in teachers indicate that vocal fatigue shows substantial short-term recovery following reduced vocal load or rest [85]. From a broader stress physiology perspective, stress responses are conceptualized as activation–recovery processes once the stressor ends, providing a theoretical framework consistent with the pattern observed in the present study [86]. On the other hand, regression results showed that vocal recovery was reduced among university professors with higher levels of stress and depression. This highlights the need to approach the treatment of dysphonia and other voice-related conditions not only through vocal hygiene or direct therapy, but also by addressing the psychosocial and emotional context. Indeed, higher relapse rates have been reported among individuals with psychogenic voice disorders who do not receive appropriate psychological support [87].

With regard to sleep quality, regression results showed that sleep-related problems were significantly associated with higher levels of stress and anxiety. Previous studies have demonstrated that stress, anxiety, and depression are closely linked to sleep quality, forming a vicious cycle in which psychological symptoms and sleep disturbances mutually intensify each other. This confirms that sleep plays a critical role in mental health, with a direct impact on the onset and persistence of these conditions [88, 89].

Furthermore, elevated stress is associated with poorer sleep quality, a relationship that has been shown to be mediated by rumination, with resilience acting as a moderator between stress and sleep [89, 90]. Studies carried out among teaching populations have also indicated that depression contributes to insomnia, and that job stress and workload are key factors related to sleep quality deterioration [91, 92]. Scientific evidence supports the existence of a bidirectional relationship between mood disorders and circadian rhythms, as these disorders are often accompanied by sleep disturbances and altered cortisol secretion, while circadian disruptions (due to jet lag, night shifts, or artificial light exposure) may exacerbate mental health conditions. Additionally, sleep is closely tied to emotional regulation. Lack of sleep negatively affects the cognitive ability to manage and process emotions, thereby increasing vulnerability to stress, anxiety, and depression [93, 94].

Regarding eating behaviour, the regression models revealed that higher levels of stress and anxiety were associated with greater emotional eating, which is considered not only a risk behaviour in itself but also a potential gateway to the development of eating disorders. Empirical evidence underscores the central role of emotions in this behaviour, as emotional eating often serves as a coping strategy to mitigate or suppress negative emotional states such as stress, anxiety, and depression [95, 96]. Supporting this, previous research has identified a strong relationship between emotional eating and psychological distress in non-clinical populations [96, 97]. Emotional states significantly influence individual eating patterns, and studies have shown that under conditions of stress or anxiety, food intake tends to increase as a mechanism of distraction or emotional escape [98, 99]. Moreover, research by Dakanalis *et al*. [100] indicates that higher levels of depressive symptoms and psychological distress are associated with an increased risk of adopting emotional eating patterns.

The present study also identified significant differences in physical activity levels and sedentary behaviour according to mental health indicators, specifically stress, anxiety, and depression. For stress and anxiety, a clear pattern emerged: groups with moderate or severe symptomatology reported lower levels of physical activity and higher sedentary time. This finding aligns with meta-analyses showing that physical activity reduces anxiety and depressive symptoms in non-clinical populations [101].

In the case of depression, although MET levels did not differ significantly between groups or follow a clear linear trend in our sample, this result should be interpreted with caution. Longitudinal studies suggest that higher physical activity levels are associated with a reduced risk of developing depression over time [102]. Conversely, a significant increase in sedentary behaviour was observed among participants with more severe depressive symptoms, suggesting that inactivity may play a particularly important role in understanding emotional distress in university faculty. Previous research has also indicated that the relationship between physical activity and depressive symptoms is not uniform. Vigorous-intensity exercise appears to be most consistently associated with reductions in depressive symptoms, while findings regarding light or moderate activity are more mixed [103]. Moreover, motivational factors, particularly introjected regulation, have been found to mediate the link between depressive symptoms and participation in moderate-to-vigorous physical activity, underscoring the influence of personal motivational contexts in this association [104].

Although no studies were found that specifically analysed this relationship in university faculty, research conducted in other educational settings has reported similar patterns. These include lower levels of physical activity and higher sedentary time among individuals experiencing stress, anxiety, and depression [105]. Taken together, these findings can be understood in light of the psychological and neurobiological mechanisms proposed in the literature to explain the benefits of physical activity. These include improvements in self-esteem, increases in brain-derived neurotrophic factor (BDNF), enhanced endorphin and serotonin release, and the modulation of neurotransmitter systems involved in emotional regulation and stress response [106, 107].

One notable strength of this study was the inclusion of 1560 university professors from thirteen Spanish universities, allowing the sample to be broadly representative at the national level. This enabled a detailed analysis of the prevalence of stress, anxiety, and depression among Spanish university faculty, as well as their associations with lifestyle habits and indicators of physical and mental health. Another strength lies in the use of a comprehensive set of validated instruments to assess psychological symptoms, well-being, lifestyle behaviours, and vocal health. These tools provided a robust and multidimensional understanding of the factors related to mental health in university teaching staff. Additionally, the inclusion of demographic, health, and lifestyle variables in the regression models allowed for a nuanced examination of the relative contributions of each factor, offering a more integrated perspective on the determinants of stress, anxiety, and depression in this population.

Nevertheless, certain limitations must be acknowledged. First, data collection relied exclusively on self-report questionnaires, which are inherently subjective and may be influenced by recall bias or social desirability. These biases may have led participants to underreport or overreport behaviours such as physical activity, dietary habits, sleep issues, or psychological symptoms, potentially attenuating or inflating some associations. However, it is important to note that the instruments used have demonstrated strong reliability and validity in similar populations. Future studies could benefit from incorporating objective or clinical assessments, such as accelerometry, voice analysis, or structured clinical interviews, to provide more precise and complementary data. Second, the cross-sectional design limits the ability to establish causal relationships. Although the study identifies significant associations between lifestyle behaviours, well-being, and psychological symptoms, the directionality of these relationships remains unclear. For instance, it cannot be determined whether unhealthy habits contribute to psychological distress or whether distress leads to poorer lifestyle choices. Longitudinal and prospective studies are needed to explore these temporal dynamics and better understand the underlying causal pathways. Third, although the sample included participants from multiple universities, convenience sampling was used. This may limit the generalisability of the findings, as those who chose to participate may differ in meaningful ways from non-respondents—possibly overrepresenting individuals with a specific interest in health or well-being. This selection bias may affect prevalence estimates and the strength of observed associations. Future research should consider probability-based sampling to enhance representativeness. Finally, although several demographic and occupational variables were included in the analyses, other potentially relevant factors such as academic discipline, teaching load, contract type, or organisational climate were not assessed. These factors could significantly influence university faculty health and should be incorporated in future studies to provide a more comprehensive understanding of contextual and organisational determinants of mental health in academic environments.

## Conclusions

The regression analysis revealed that stress, anxiety, and depression were negatively associated with mental well-being and positively associated with emotional exhaustion and physical vocal discomfort. Sleep problems and emotional eating were also linked to higher levels of stress and anxiety, whereas improvement in voice symptoms was associated only with lower stress levels. Physical well-being emerged as a significant predictor exclusively for stress. Importantly, demographic factors also proved relevant, with women and younger faculty members reporting higher levels of distress. This finding aligns with previous research and underscores the need to consider sociodemographic characteristics when designing interventions to address stress, anxiety, and depression in university faculty. Additionally, significant associations with sedentary behaviour were observed, reinforcing the importance of lifestyle factors in the emotional health of university educators. Although the relationship between physical activity and depressive symptoms did not follow a linear pattern in this sample, sedentary time consistently correlated with poorer mental health, suggesting that reducing sedentary behaviour may represent a valuable intervention target. Taken together, these findings highlight the multidimensional nature of the factors contributing to psychological well-being and distress among university teaching professionals.

The implications of this study point to the need for universities and policy-makers to develop comprehensive strategies aimed at promoting psychological well-being in academic staff. The observed prevalence of stress, anxiety, and depression demands action, not only due to their impact on faculty health but also because of their potential consequences for teaching quality, organisational climate, and institutional sustainability. To effectively reduce psychological distress and foster healthier, more sustainable academic environments, a multilevel approach is required. This should address individual, organisational, and behavioural factors through well-being programmes, training in coping skills, recognition and support systems, work–life balance policies, and the promotion of healthy lifestyles.

## Availability of Data and Materials

The data analyzed was available on the request for the corresponding author.
